# Poly-γ-glutamic acid promoted maize root development by affecting auxin signaling pathway and the abundance and diversity of rhizosphere microbial community

**DOI:** 10.1186/s12870-022-03908-y

**Published:** 2022-11-10

**Authors:** Haizhen Ma, Panpan Li, Ning Xiao, Tao Xia

**Affiliations:** 1 State Key Laboratory of Biobased Material and Green Papermaking, Qilu University of Technology (Shandong Academy of Sciences), Jinan, Shandong PR China; 2 School of Bioengineering, Qilu University of Technology (Shandong Academy of Sciences), Jinan, 250353 Shandong PR China

**Keywords:** Maize, γ-PGA, Auxin signaling, Roots development, Plant growth promoting bacteria

## Abstract

**Background:**

The root systems of higher plants play an important role in plant growth and development. In our present study, it was found that poly-γ-glutamic acid (γ-PGA), an environmentally friendly biomacromolecule, significantly improved root development in maize.

**Results:**

After treatment with γ-PGA for 7 days, the fresh weight of maize roots was significantly increased and the differences between γ-PGA treated group and control group were mainly caused by the number (higher by 71.87% compared to the control) and length of lateral roots. RNAseq and RT-PCR analyses showed that γ-PGA treatment upregulated the expression of genes related to the synthesis of auxins and auxin signal in maize roots. In addition, γ-PGA promoted the accumulation of plant growth-promoting bacteria, such as *Azospirillum, Azohydromonas, Ramlibacter,* and *Sphingobium* (Proteobacteria), *Streptomyces* (Actinobacteria), *Parasegetibacter* (Bacteroidete*s*), and *Gemmatimonas* (Gemmatimonadetes) in rhizosphere soil and the secretion of auxins. The results of this study deepened our understanding of the effects and mechanism of γ-PGA on maize root development, and as well as highlighted the possibility of using γ-PGA to improve crop growth and soil environment.

**Conclusions:**

γ-PGA promotes early growth and development of maize roots by inducing the secretion and accumulation of auxin in roots and in rhizosphere soil, and increasing the abundance of plant growth promoting bacteria.

**Supplementary Information:**

The online version contains supplementary material available at 10.1186/s12870-022-03908-y.

## Background

Maize (*Zea mays* L.) is an important crop, which is used as animal feed, human food and bioethanol production. The complex root system of maize facilitates the uptake of water and nutrients and the anchorage of maize in the soil, and deeply affect the growth and development of maize [1]. The root system of maize can be divided into embryonic root system and post-embryonic root system. Embryonic root system includes a single primary root and a variable number of seminal roots. Post-embryonic root system contains crown roots, brace roots and lateral roots emerged from all major root types [2]. In all maize root types, lateral roots are initiated from phloem pole pericycle and endodermis cells [3].

Root architecture encompasses the density of lateral roots (LR) and the root branching pattern, which comprise the lateral and adventitious roots. In maize, root branching is an important aspect for root structure development. Well-branched roots increase the surface area for the absorption of water and nutrients. Adventitious roots, including aerial roots and root cap formed by underground nodes, anchor the plant and facilitate the absorption of water and mineral elements at the mature stage of maize. The development of lateral roots can start from the columnar sheath cells of the primary roots, the seminal roots, and the underground nodes of roots [4, 5]. The formation of lateral roots is a critical element of the root system because it affects the absorption of deeper water and nutrients. Lateral roots arise from the xylem pole pericycle cells, which undergo a series of divisions to form LR primordium before developing into lateral roots [6].

Auxins regulate the lateral and vascular root development and the architecture of roots [7–9], which mediates a variety of physiological processes and has long been known to promote lateral root formation [10]. Auxin synthesis, transport, and signaling pathways are triggered during the formation of lateral root (LR) [11]. Auxins are primarily synthesized via the Indole-3-pyruvic acid (IPA) pathway. In this pathway, tryptophan (TRP) is firstly converted to IPA by transaminases of the TAA family, before IPA is converted to IAA by YUC enzyme. YUC are enzymes usually located on the membrane of endoplasmic reticulum [12]. Synthesis/function of auxins is mainly regulated by the Transport Inhibitor Response 1 (TIR1) protein, members of the Auxin F-Box (AFBs) family, AUXIN/INDOLE-3-ACETIC ACID (Aux/IAA) proteins, and Auxin Response Factor (ARF) proteins [13, 14]. The TIR1 is an auxin receptor and also part of the E3 ubiquitin ligase complex SCF (TIR1). At low intracellular concentrations of auxins, the transcription of the Aux/IAA proteins was repressed by interacting with ARF proteins [15]. High levels of auxin promote the binding of Aux/IAA proteins to auxin receptor protein TIR1, inducing the ubiquitination and degradation by the 26S proteasome [16–18]. ARF proteins are then released from Aux/IAA complex, and their activation promotes the transcription of auxin-responsive target genes [19]. Different TIR1/AFB-Aux/IAA combinations may induce different transcriptional responses [8]. Auxin promotes LR development through SLR4/ARF7-ARF9 signal module [20]. The *rum1* gene encodes an Aux/IAA protein ZmIAA10 which was required for the initiation of embryonic seminal and post-embryonic lateral root initiation in primary roots of maize [21]. RUM1 could interacts with the transcriptional activators ZmARF25 and ZmARF34. The mutated rum1 protein cannot interact with SCF^TIR1^ E3 ubiquitin–ligase complexes which prevents its ubiquitin-mediated proteasomal degradation and resulting in the constitutive repression of downstream gene expression [3].

Poly-γ-glutamic acid (γ-PGA) is a nontoxic, water-soluble, biodegradable and environmentally friendly biopolymer, composed of D/L-glutamic acid monomers and produced through *Bacillus subtilis-*mediated fermentation [22, 23]. γ-PGA can be applied in food, medicine, cosmetics, and agricultural fields based on its variable molecular weight [24]. Recent studies have shown that γ-PGA plays an important role in plant growth and development and, thus, is a promising supplement in agricultural fertilizers. γ-PGA significantly increases the yield of several crops, including cucumber, Chinese cabbage, wheat, and rapeseed [25, 26]. These studies made an effort to reveal the promotional effect of γ-PGA on plant growth from the perspective of plant nitrogen metabolism [27]. The root is an integral tissue of the plant and plays the most important role in the absorption and utilization of nutrients [28]. A few studies have found that γ-PGA could improve root biomass [27]. However, how γ-PGA promotes root growth remains unclear. Herein, we investigated the effect of γ-PGA on the synthesis and signal transduction of auxins in maize roots and the rhizosphere soil microbial community, in order to comprehensively understand the effect and mechanism of γ-PGA promoting maize root development.

## Results

### Exogenous application of γ-PGA could promote the development of maize roots

**Fig. 1:**
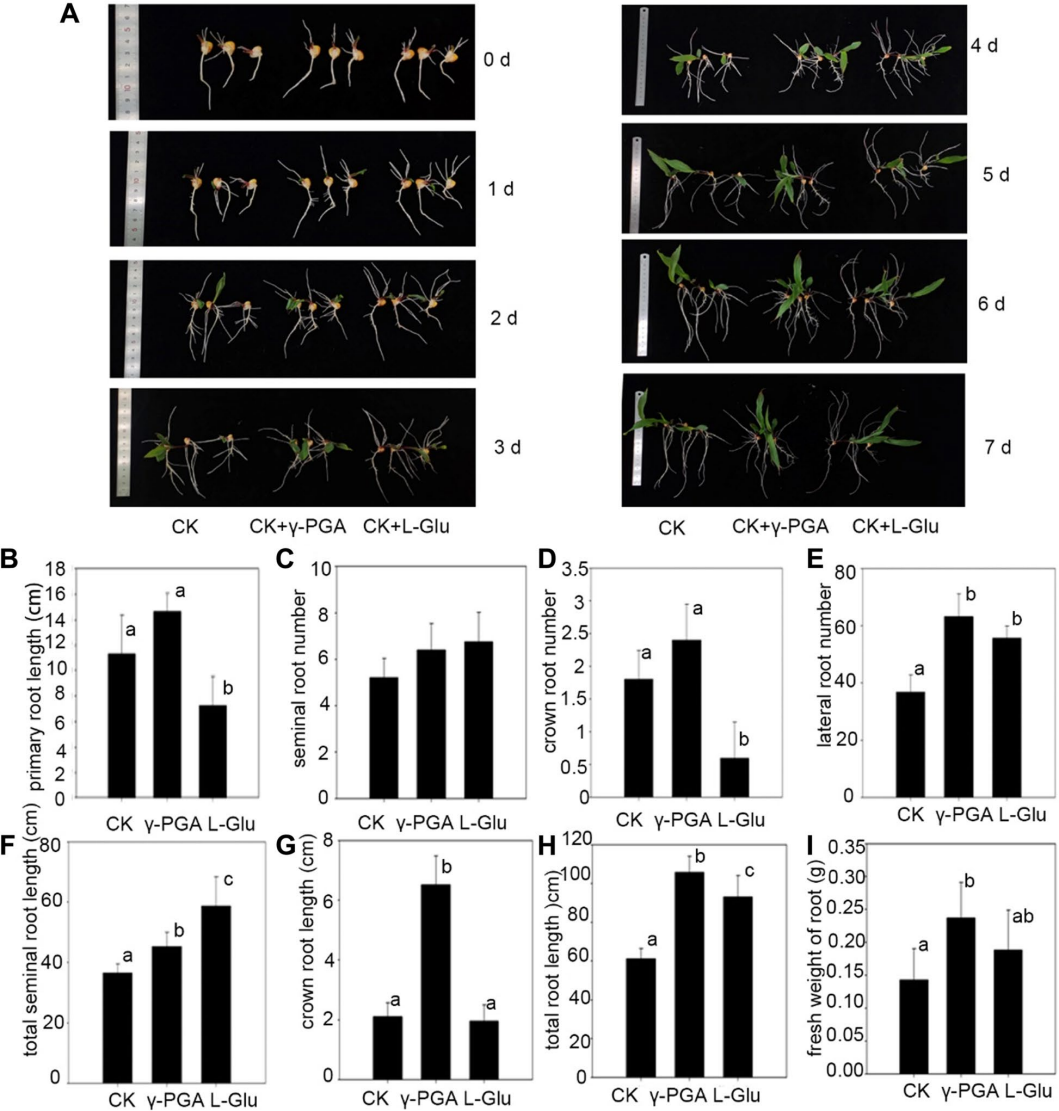
The effect of γ-PGA on the appearance of maize roots, IAA content in roots and rhizosphere soil, and urease activity of rhizosphere soil. **A** Phenotypes of maize after γ-PGA (10 kDa, 50 mg/L) and L-Glu (50 mg/L) treatment. **B** The primary root length of maize with γ-PGA, L-Glu treatment, and control maize (CK). **C-E** The seminal root, crown root and lateral root number of maize with γ-PGA, L-Glu treatment, and control maize (CK). **F-H** The total seminal root, crown root and total root length of maize with γ-PGA, L-Glu treatment, and control maize (CK). **I** The fresh weight of maize roots with γ-PGA, L-Glu treatment, and control maize (CK). Values are means ± sd (*n* = 5 repeats). Significant differences are indicated by different letters (*P* ≤ 0.01)

In order to analyze the effect of γ-PGA on the development of maize root system, the seedlings germinated after 5 days were treated with γ-PGA (Fig. 1A). Previously, the effects of different molecular weight γ-PGA on maize growth and drought resistance, were studied and found that low molecular weight γ-PGA could promote the growth and drought resistance of maize (Fig. S1). Therefore, γ-PGA of 10 kDa was used to study its effects on maize root development in this study. The results showed that there were obvious lateral roots on the primary root after γ-PGA treatment for 1 day (Fig. 1A). After γ-PGA treatment for 7 days, the number and length of roots of different types of maize were counted (Fig. 1B-I). The number of seminal roots and crown roots did not significantly change between γ-PGA treated group and control group (Fig. 1C, D), but the number of lateral roots in γ-PGA treated group was significantly higher than that of the control group by 71.87% (Fig. 1E). The total root length of γ-PGA treated group was 72.58% higher than that of the control group, the differences between γ-PGA treated group and control group were mainly caused by the number and length of lateral roots (Fig. 1H). In addition, the length of seminal roots was 24.19% longer than that of the control group, and the length of crown roots was 210% longer than that of control group (Fig. 1F, G). We also treated the roots used the same concentration of L-Glutamate (L-Glu) (Fig. 1A). The results showed that the primary root length of maize after L-Glu treatment decreased significantly (35.84% less than that of the control group and 65.22% less than that of γ-PGA treated group) (Fig. 1B). However, after L-Glu treatment, the number of seminal roots increased (29.80% more than the control group and slightly higher than the γ-PGA treated group, but the difference was not significant) after L-Glu treatment (Fig. 1C). The number of lateral roots of L-Glu treatment group was 51.08% more than that in control group, but 12.10% less than that in γ-PGA group (Fig. 1E). However, the length of seminal roots in L-Glu treatment group was 60.86% longer than that of the control group and 29.52% longer than that of γ-PGA treatment (Fig. 1F). The total root length of L-Glu group was 51.73% more than that of control group, but 12.08% less than that of γ-PGA group (Fig. 1H). Finally, the fresh weight of maize roots after 7 days‘treatment was calculated. The results showed that the fresh weight of maize roots after γ-PGA treatment was also significantly increased (Fig. 1I). Therefore, the promotion of the root development after γ-PGA treatment was caused principally by the number and length of lateral roots.

### Exogenous γ-PGA induced the secretion and accumulation of auxin in maize roots

**Fig. 2:**
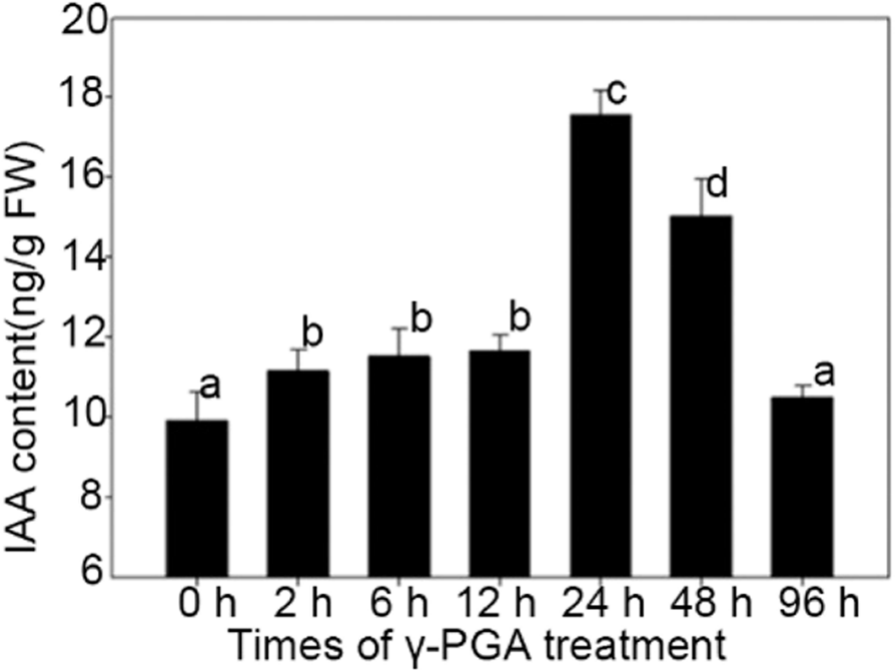
The effect of γ-PGA treatment on IAA content in maize roots. The effect of γ-PGA treatment on IAA content in maize roots after 0 h, 2 h, 6 h, 12 h, 24 h, 48 h, and 96 h. Values are means ± sd (*n* = 3 repeats). Significant differences are indicated by different letters (P ≤ 0.01)

It’s reported that auxin plays an important role in the development of plant roots. In order to explain the mechanism of γ-PGA promoting maize root development, the contents of auxin in maize roots after 0 h, 2 h, 6 h, 12 h, 24 h, 48 h, and 96 h of γ-PGA treatment were measured. The results showed that γ-PGA could induce the synthesis and accumulation of auxin (increased by 12.51%) in roots just after 2 h of γ-PGA treatment. After 24 h and 48 h of γ-PGA treatment, the auxin content in maize roots increased by 77.09 and 51.70% (Fig. 2). The results indicated that exogenous γ-PGA could induce the accumulation of auxin in maize roots.

### γ-PGA promotes the synthesis and accumulation of auxins and auxin-response genes

**Fig. 3:**
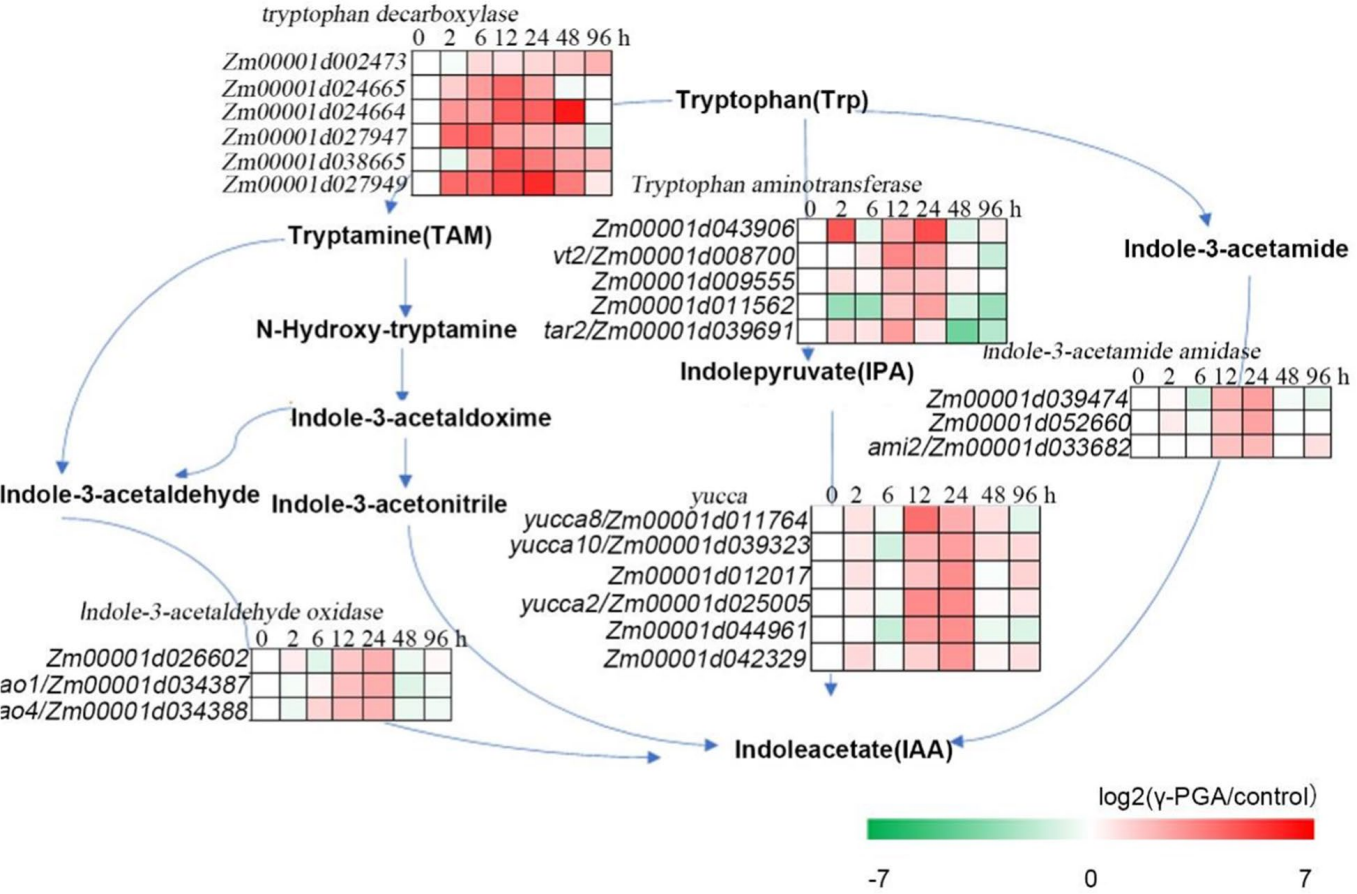
The effect of γ-PGA on the expression of genes related to auxin synthesis. The maize roots were treated with γ-PGA, and the expression of genes related to auxin synthesis in the roots after treatment for 0 h, 2 h, 6 h, 12 h, 24 h, 48 h, and 96 h. And the maize without treatment was used as the control. Expression levels of these genes were analyzed by real-time RT-PCR, fold changes in transcripts were calculated by 2^-ΔΔCt^ method with *ZmTub* as an internal control. And the heatmaps of these genes were made using the value of log2 (γ-PGA/control) of the relative expression level

**Fig. 4:**
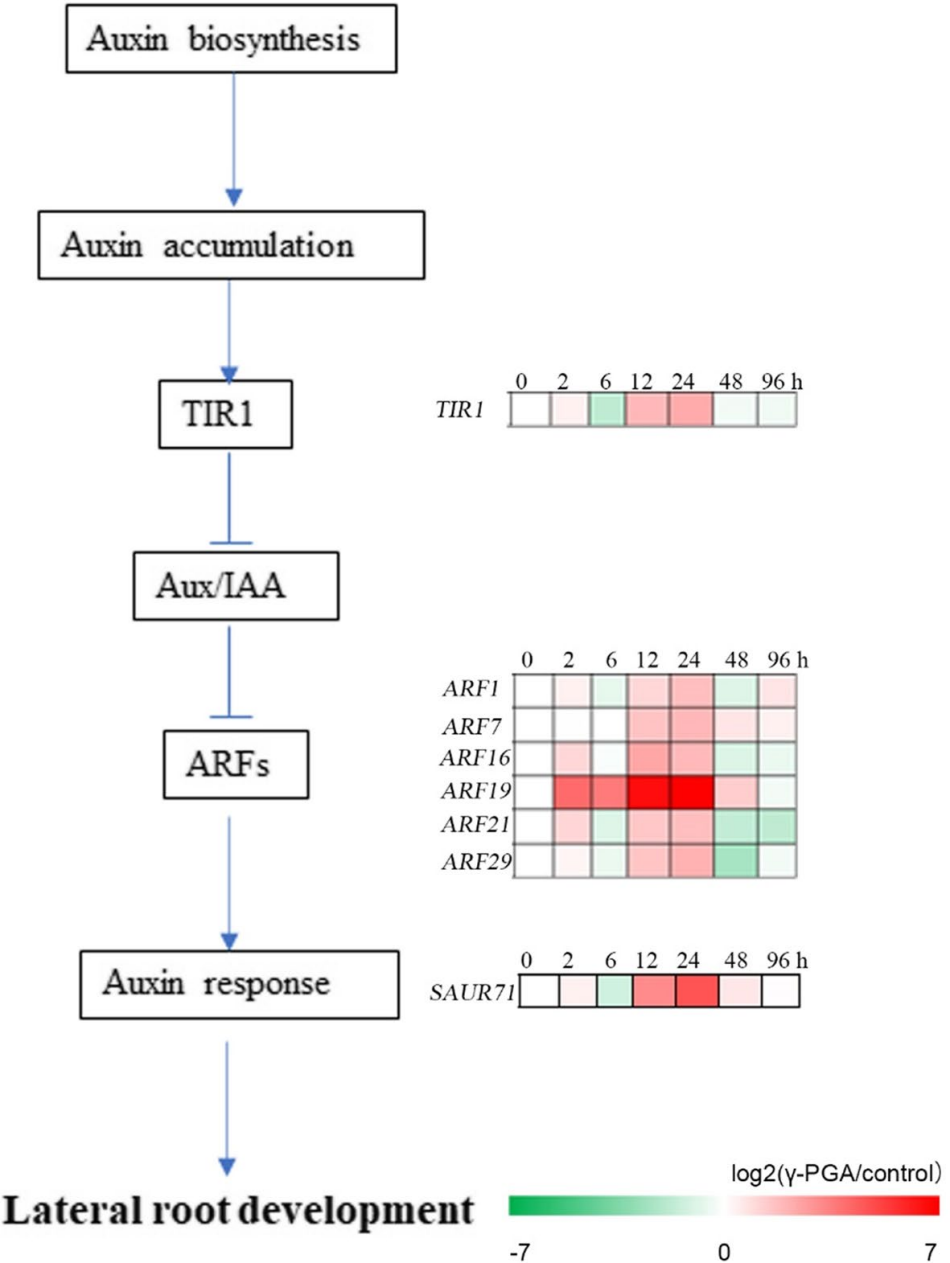
The effect of γ-PGA on the expression of genes related to the biosynthesis of auxins. The maize roots were treated with γ-PGA, and the expression of genes related to auxin biosynthesis in the roots after treatment for 0 h, 2 h, 6 h, 12 h, 24 h, 48 h, and 96 h. And the maize without treatment was used as the control. Expression levels of these genes were analyzed by real-time RT-PCR, fold changes in transcripts were calculated by 2^-ΔΔCt^ method with *ZmTub* as an internal control. And the heatmaps of these genes were made using the value of log2 (γ-PGA/control) of the relative expression level

The results of RNAseq of roots treated with and without γ-PGA performed previously revealed that γ-PGA upregulated the expression of genes related to the synthesis of auxins (Supplementary Fig. S2). In tryptophan-dependent IPA auxin synthesis pathway, γ-PGA treatment upregulated the expression of 7 TAA1-encoding genes in maize roots. γ-PGA treatment dysregulated the expression of 9 genes encoding YUCCA protein, in which 7 were over-expressed and 2 were under-expressed. Also, γ-PGA treatment dysregulated the expression of 12 DEG genes in tryptamine synthesis pathway, in which 11 were upregulated, whereas only one gene was down-regulated. γ-PGA treatment also dysregulated the expression of 6 genes in indole-3-acetamide pathway, in which 3 were down-regulated, 3 were upregulated (Supplementary Fig. S2). Furthermore, we also analyzed the expression of these genes in maize roots of γ-PGA treatment for 0 h, 2 h, 6 h, 12 h, 24 h, 48 h and 96 h (Figs. 3 and 4). The results showed that the expression of these genes was induced by γ-PGA. In addition, 6 genes for ARF, 1 gene for TIR1, and 1 gene for SAUR71 were also induced by γ-PGA in auxin signaling pathway (Fig. 4). Overall, these results suggested that γ-PGA promoted root growth via the auxin synthesis and auxin signaling pathways (Figs. 3 and 4).

### γ-PGA increased the content of auxin and urease activity of maize rhizosphere soil

Since maize rhizosphere soil was in close contact with the roots, the urease, which is closely related to soil nitrogen transformation, and IAA contents of maize rhizosphere soil, was also detected. It was found that γ-PGA treatments increased the urease activity in the rhizosphere of maize by 41.12%, whereas the IAA contents in the soil after γ-PGA treatments increased by 16.83% (Table 1).

**Table 1:** Effect of γ-PGA on the contents of IAA and Urease activity of maize rhizosphere soil

Drought	IAA mg/g DW)	Urease activity (μg NH_3_-N/g/24 h)
0 mg/L γ-PGA	0.708 ± 0.001	917.677 ± 34.415
50 mg/L γ-PGA	0.828 ± 0.015**	1295.056 ± 74.306**

Values are means ± sd (n ≥ 3 repeats). Significant differences are indicated by asterisks (**, *P* ≤ 0.01)

### γ-PGA changed the microbial community in maize rhizosphere soil

**Fig. 5:**
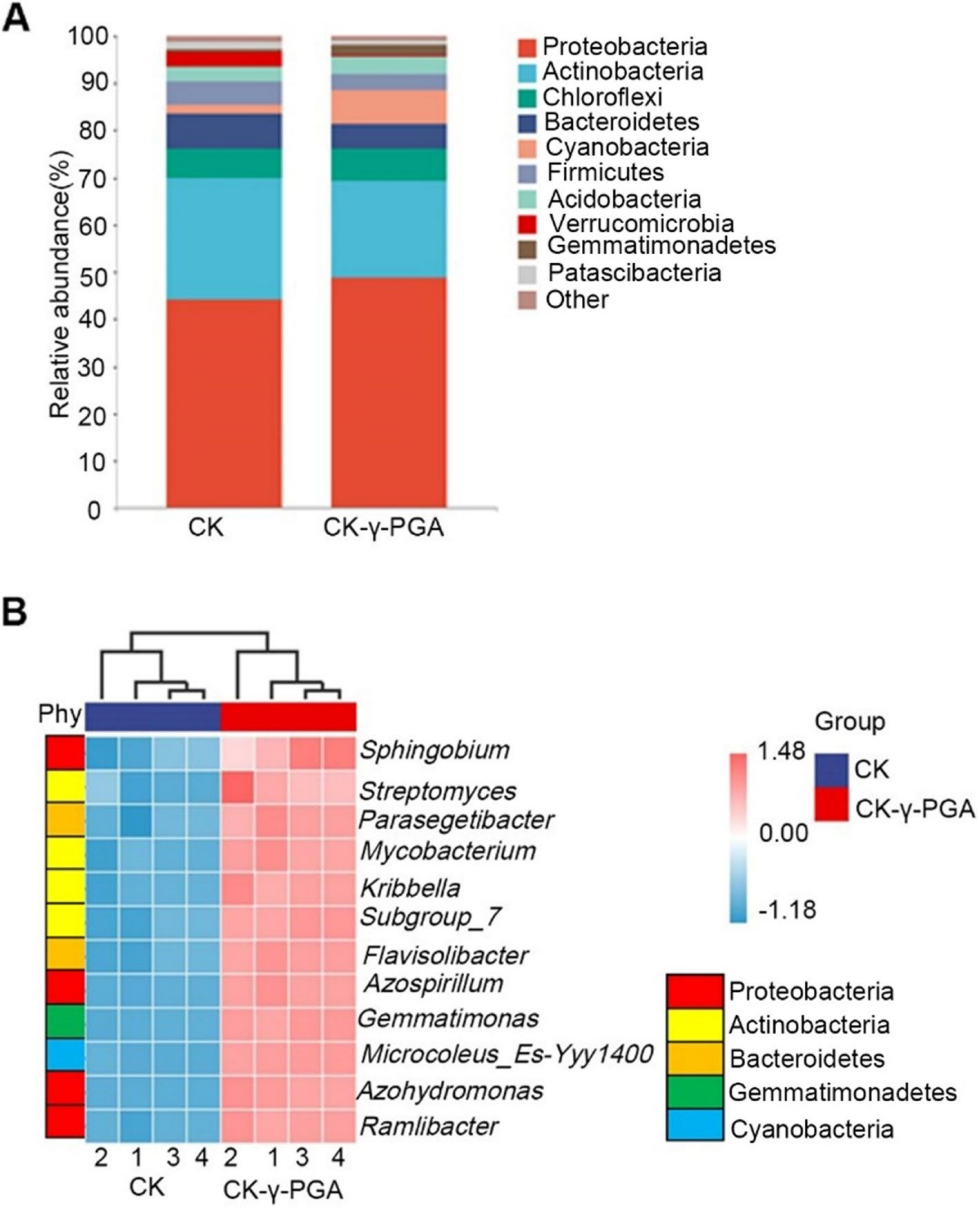
The effect of γ-PGA on the relative abundance and diversity of beneficial bacteria in the rhizosphere of maize. **A** The effect of γ-PGA on the relative abundances of bacterial communities at the phylum level in the rhizosphere of maize. **B** The bacteria species in the maize rhizosphere are most influenced by γ-PGA treatment

The effect of γ-PGA on the diversity and abundance of the bacterial community in the maize rhizosphere was analyzed using high-throughput sequencing of the 16S rDNA region. NMDS (stress = 0.000108) of the Bray-Curtis UniFrac distance ordinations were also performed (Supplementary Fig. S3A), and the results indicated that the bacterial community composition of rhizospheric soil with γ-PGA application shifts compared with that of the soil without γ-PGA. The communities in maize rhizospheric soil with γ-PGA were grouped together and significantly separated from those in soil without γ-PGA. The obtained high-quality sequences belonged to 36 phylum，among which the main phylum was Proteobacteria, followed by Actinobacteria, Chloroflexi, Bacteroidetes and Cyanobacteria. Although the abundance of bacterial community changed after the addition of γ-PGA，the predominant phylum were similar. There was no difference in species composition among the γ-PGA-treated and non-treated groups; Compared to the control, the relative abundance of Proteobacteria, Acidobacteria, Cyanobacteria and Gemmatimonadetes were higher in soil added γ-PGA (Fig. 5A). The LEfSe analysis (LDA ≥ 3.35) had been used to obtain the species with the most significant variation (Supplementary Fig. S3B). At phylum level, γ-PGA treatment increased the abundance of Proteobacteria, Acidobacteria, Cyanobacteria and Gemmatimonadetes, Deltaproteobacteria and Gammaproteobacteria at class level (Fig. S2B). At genus level, γ-PGA treatment increased the abundance of *Azospirillum, Azohydromonas, Ramlibacter and Sphingobium* (Proteobacteria), *Subgroup_7*, *Mycobacterium*, *Kribbella* and *Streptomyces* (Actinobacteria), *Flavisolibacter* and *Parasegetibacter* (Bacteroidetes), *Gemmatimonas* (Gemmatimonadetes), and *Microcoleus_Es-Yyy1400* (Cyanobacteria) in the maize rhizosphere (Fig. 5B).

## Discussion

### The promotion of the root development by γ-PGA may be not entirely caused by L-Glu which was generated after γ-PGA degradation

Our study showed that γ-PGA significantly improved the growth of maize, which is consistent with previous findings. In this project, we mainly focused on the effect of γ-PGA on root development and found that γ-PGA can promote the growth of maize lateral roots. At the same time, γ-PGA significantly increased the biomass of maize roots, promoted the growth of roots, enhanced the ability of maize to absorb nutrients and promoted the growth of plants. In order to further explain the mechanism of γ-PGA promoting root growth, we also determined the auxin content and the expression of the auxin related genes in maize roots. The results showed that exogenous γ-PGA could induce the secretion and accumulation of auxin in maize root, which had not been reported in the literature.

Studies have shown that L-Glutamate (L-Glu) can not only promote plant growth as a nitrogen nutrient, but also serve as a plant signal molecule. Glutamate can be involved in calcium signaling and root development. For example, L-Glu can inhibit the growth of primary roots of *Arabidopsis thaliana* and stimulate the growth of lateral roots near the primary root tip. Other amino acids, such as Gln and D-Glu, have no similar effect [29]. In addition, MEKK1 is involved in glutamate signaling pathway, which is involved in inducing changes in the root structure of *Arabidopsis* [30]. Exogenous L-Glu can not only support the growth of rice seedlings as nitrogen, but also induce the expression of a series of genes to regulate the development of rice roots [31]. Since γ-PGA is easily degraded into L-Glu and D-Glu, in order to prove whether the promotion of maize root development is the effect of exogenous γ-PGA or its degradation product L-Glu, we treated maize roots with the same concentration of L-Glu and compared it with γ-PGA. The results showed that both γ-PGA and L-Glu could change the development of maize roots, but the changes of different types of maize roots were different. The effect of L-Glu on maize root was not as good as γ-PGA. Many studies have shown that γ-PGA can significantly promote the growth of plant roots. γ-PGA treatment can improve the physical and chemical properties of soil, increase soil microbial biomass and microbial activity, and promote the absorption of nutrients by plants. The results showed that although both treatments increased root biomass, the effects on different types of maize roots were different. Previous studies have shown that γ-PGA may act as an N source to promote plant growth, and its mechanism of promoting root development may be related to L-Glu. In this study, maize roots were directly treated with the same concentration of γ-PGA and L-Glu respectively, and the culture medium was changed every day to prevent the degradation of γ-PGA as much as possible. The results showed that the difference between γ-PGA treatment group and control group was mainly caused by the number and length of lateral roots. However, the root length of L-Glu treatment decreased significantly and the number of seed roots became variable, while the number of lateral roots also increased, but the increase range was less than that of γ-PGA treatment. The total root length of L-Glu treatment group was 51.73% higher than that of control group, but 12.08% lower than that of γ-PGA treatment group. The results indicated that the mechanism of γ-PGA promoting maize root development may be different from that of L-Glu.

### γ-PGA may promote root development by inducing auxin synthesis

As sessile, plants rely on hormones to coordinate different stages of development and increase the plasticity of plant development in a changing environment. For example, under abiotic stresses such as drought or nutrient deficiencies, plants will induce root elongation and root structure changes, so as to penetrate deeper into the soil and obtain nutrients and water. Plant hormones play an important role in the regulation of root structure [32]. Among them, auxin regulates the division and differentiation of root meristem cells, the growth of primary roots and the development of lateral roots.

Plants absorb water and nutrients through roots, which support the growth and development of plants. Thus, the change in root morphology deeply affects the growth and development of plants. Auxins regulate plant lateral root and vascular development, thus regulating root architecture [7–9]. Indole-3-pyruvic acid (IPA) pathway is the main pathway for synthesizing auxins. In this pathway, tryptophan (TRP) is firstly converted to IPA, catalyzed by transaminase belonging to TAA family. IPA is then converted to IAA by YUC enzyme. YUC is a family of enzymes usually attached to the membrane of endoplasmic reticulum [12]. Studies have confirmed the presence of YUC protein in plant root tissue [33]. In this study, it was found that 7 DEGs encoding TAA protein were upregulated, 9 genes encoding YUC protein dysregulated in maize roots with γ-PGA treatment. Of these, the expression of 7 DEGs was upregulated, 2 DEGs was downregulated. In addition，γ-PGA treatment upregulated the expression of 11 genes related to the tryptamine (auxin) synthesis pathway and of 3 genes that regulate the activities of Indole-3-acetamide auxin pathway, which are all related to the synthesis of auxins (Fig. 3). Many studies have shown that auxin plays an important role in lateral roots (LR) formation, especially in LR initiation and primordial development [34, 35]. Auxin signaling pathway, mediated by the Aux/IAA and ARF transcription factors, is required during LR initiation process [4]. During this process, auxin signals are transmitted to peripheral cells to promote the degradation of Aux/IAAs (such as IAA14/SLR). The degradation of Aux/IAAs initiates the development of lateral roots through the SCF^TIR1/AFBs^ complex and 26S proteasome. This activates the ARF7/ARF19 pathway, which promotes the expression of target genes such as *LBD16/ASL18* and *LBD29/ASL16* that initiates the development of LR [36]. In the present study, we also found that γ-PGA treatment induced the expression of 6 *ARF*, 1 *TIR1*, and 1 *SAUR71* genes, all of which were related to the synthesis of auxins (Fig. 4). The results showed that γ-PGA treatment significantly up-regulated the expression of genes involved in auxin synthesis and signaling pathways within a few hours, and the increased auxin concentration rapidly induced the initiation of lateral roots [37], but auxin was always in homeostasis in plants. Plants maintain auxin homeostasis by coordinating the biosynthesis, transport, inactivation of auxin [38]. Therefore, γ-PGA may constantly induce the accumulation of auxin, and the up-regulated auxin concentration must induce other unknown pathways that negatively regulate auxin biosynthesis to keep the auxin homeostasis, which may be the reason for the decrease of auxin concentration after 96 h of γ-PGA treatment.

### γ-PGA may promote root development by altering the composition of microorganisms in soil rhizosphere

Plant development affects the interaction of rhizosphere microbial communities [39]. Metabolites secreted by plant roots will alter the soil microbial composition. Primary and secondary metabolites produced and exuded by plants can selectively promote or inhibit specific microbial communities [40, 41]. Conversely, microbial activities also influence plant development and response to environmental factors [42, 43]. Soil microorganisms can stimulate the growth of lateral roots and root hairs, and further improve water and nutrient uptake. In addition, soil microorganisms can also promote plant growth and regeneration. Soil microbes can affect both internal and external processes that supporting plant growth and development.

Under stress conditions, well-developed root structures facilitate the recruitment of beneficial microorganisms from microbiome-rich topsoil. In turn, microorganisms can produce or alter phytohormone levels in the rhizosphere or plants, thereby influencing plant development and stress responses [44, 45]. Numerous hormones (IAA, ABA, CKs, Gas, and ET) have been isolated from the growth medium of soil microorganisms. Hormone producing microorganisms, such as plant growth-promoting bacteria (PGPBs) and plant growth-promoting fungi (PGPFs)，are usually non-pathogenic and even beneficial to plants [46]. Studies have shown that beneficial microorganisms can produce hormones or activate the synthesis of plant hormones, which changes the structure of plant roots to provide a habitat for the microorganisms. For example, PGPBs affect the division and differentiation of root cells, thus changing the root structure [47]. *Bacillus megaterium* promotes the development of root structure through plant CK signaling pathway, which is independent of ET and auxin pathways [48]. In this study, we found that γ-PGA increases the synthesis of IAA (auxin) in maize rhizosphere. PGPBs synthesize IAA in maize rhizosphere. Although γ-PGA had no effect on the composition of dominant bacteria species, it strongly influenced their relative abundance. γ-PGA increased the relative abundance of Proteobacteria at phylum level. And previous studies have shown that some of Proteobacteria were participated in nitrogen fixation [49]. At the genus level, exogenous γ-PGA significantly increased the abundance of *Azospirillum, Azohydromonas, Ramlibacter, Sphingobium* of Proteobacteria, *Streptomyces* of Actinobacteria, *Parasegetibacter* of *Bacteroidetes* and *Gemmatimonas* of Gemmatimonadetes, most of which are PGPBs [50–53]. *Azospirillum*, *Azohydromonas* and *Sphingobium* are nitrogen-fixing bacteria, which can promote nitrogen absorption and plant growth [51, 54]. It was also reported that *Azospirillum Brasilense*, a PGPR of *Azospirillum*, could secretes IAA, nitric oxide, carotenoid and several cell surface components that promote plant growth [55, 56]. *Streptomyces* enhance plant growth and inhibits the growth of phytopathogens [57, 58]. *Parasegetibacter*, also as a PGPR, had important potential in promoting plant growth [59]. In addition, we found that γ-PGA treatment increased the urease activity in maize rhizosphere soils (Fig. 1H). Urease in the soil catalyzes the conversion of nitrogen to NH_3_, NH_4_^+^ and CO_3_^2-^, participates in nitrogen transformation by catalyzing the conversion of nitrogen to NH_3_, NH_4_^+^, and CO_3_^2−^ through urea hydrolysis and thus providing nutrients for the plants. These findings imply that γ-PGA altered the microbial diversity in soil rhizosphere and increased the abundance of bacteria promoting root development.

However, the effects of γ-PGA treatment on maize rhizosphere microbial community under normal growth conditions and drought conditions are different (Fig. S4). γ-PGA treatment mainly enriched some drought-resistant plant growth promoting bacteria, such as Actinobacteria, Chloroflexi and Cyanobacteria under drought stress [60]. Actinobacteria and Chloroflexi were reported to be the most prominent phyla under drought conditions [61]. Under normal irrigation conditions, the application of γ-PGA significantly enriched Proteobacteria, Acidobacteria, Cyanobacteria and Gemmatimonadetes, which can promote nitrogen absorption and auxin secretion, thus promoting the development of plant roots. In the rhizosphere soil without γ-PGA treatment, the amounts of microorganisms decreased significantly after drought stress, which was consistent with the previous report that drought may be the abiotic stress with the greatest impact on soil biological community, usually resulting in a significant reduction of microbial biomass [62].

## Conclusions

**Fig. 6:**
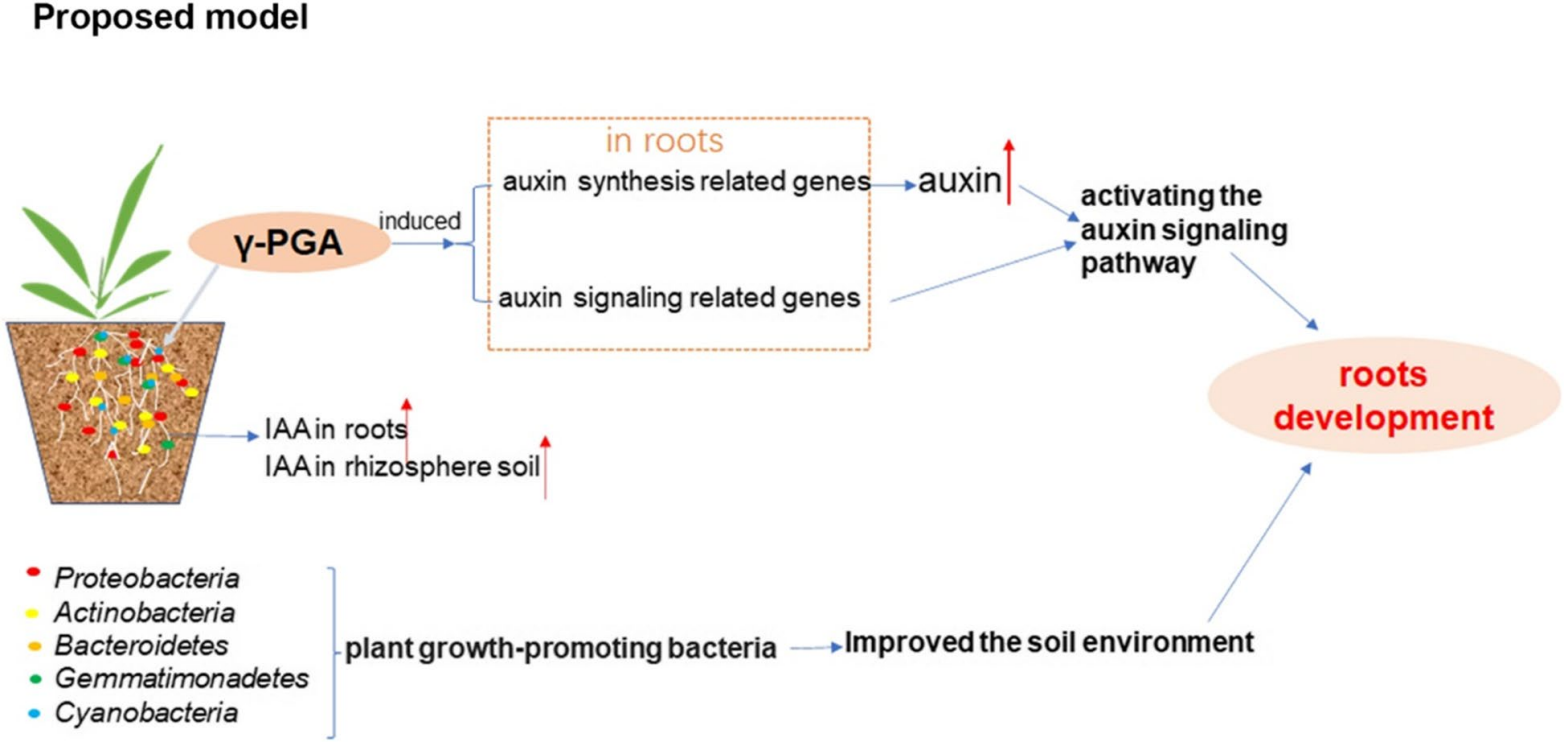
The proposed model of how γ-PGA affects the development of maize roots

Exogenous application of γ-PGA could enhance the abundance of PGPR in maize rhizosphere soil, increase the content of auxin in maize root and rhizosphere soil and promote the development of maize roots (Fig. 6). This study provides a more comprehensive understanding of the role and mechanism of exogenous application of γ-PGA in improving root development, and is conducive to its application in agriculture.

## Materials and methods

### Plant materials

KN5585 maize seeds were sown in a soil box (10 cm*10 cm*10 cm). When the seedlings grew to 1-leaves, the seedlings were watered with solution 50 mg/L γ-PGA of different molecular weight (0, 10, 100,700, 1000, and 2000 kDa), and grown in greenhouse (28 ± 2 °C under nature light, and 25 ± 2 °C at night). At three-leaf stage, all seedlings were exposed to drought stress treatment by stopping watering to select the most suitable molecular weight of γ-PGA. KN5585 maize seeds were sterilized using 75% ethanol and germinated for 4 days in the dark at 28 °C on a moisturized filter paper in a sterile culture dish (diameter: 12.5 cm). The germinated seeds were transferred into a flask 15 cm high and 7 cm wide. Culturing was performed at 28 °C /25 °C (16 h light/8 h dark). The seedlings germinated for 6 days were treated with γ-PGA solution (10 kDa, 50 mg/L). The γ-PGA solution was replaced every day to prevent it from degrading and was aerated with a mini air pump. The number and the length of the main types of the maize roots were determined after 6 days. Finally, the plants were also examined phenotypically, and the fresh weight of the roots were also determined.

### Determination of auxin content in maize roots

The IAA content of roots was measured at 0 h, 2 h, 6 h, 12 h, 24 h, 48 h and 96 h. About 1.0 g of each sample was rapidly frozen in liquid nitrogen and homogenized to powder. IAA was extracted and quantified according to the manufacturer’s instructions (Wuhan Metware Biotechnology Co., Ltd., Wuhan, China). IAA was quantified using LC-MS/MS system. The content of IAA was determined by external standard method and three biological replicates were carried out.

### RNA extraction and real-time RT-PCR

The total RNA from the maize roots after treatment with γ-PGA after 0 h, 2 h, 6 h, 12 h, 24 h, 48 h, and 96 h was extracted using the HiPure RNA Kit (Magen, Guangzhou, China). The RNA (2 μg) was reverse transcribed into cDNA using a Reverse transcription kit (TAKARA). The genes of interest and the internal control (*ZmTub*) were amplified using the SYBR Green I Master Mix (Roche, Indianapolis, USA) in the LightCycler 480 (Roche, USA) platform. Each gene was amplified in triplicate. The amplification conditions included initial denaturation at 95 °C for 5 min, subsequent denaturation through 40 cycles at 95 °C for 10 s, annealing at 60 °C for 10 s, and elongation at 72 °C for 20 s. The relative amplification of genes was calculated using the 2^-ΔΔCT^ method. Differences between different groups were analyzed using ANOVA. Data were analyzed using the SPSS software, version 20. The primers used in this research are shown in Supplementary Table S1.

### Determination of auxin content and urease activity in maize rhizosphere soil

The determination of the auxin content in maize rhizosphere was according to the previous method [63]. The content of IAA was determined with High-performance liquid chromatography (HPLC, L-2000, Hitachi), using the Extend-C18 column size (5 μm* 4.6 mm*150 mm), the absorbance of IAA was 280 nm. The urease activity was determined as previously described by Wang [64]. And five biological replicates were performed.

### Bacterial community analysis of maize rhizosphere soil

Maize (KN5585) seeds were sown in a cubit soil box measuring 10 cm. After germination, the seedlings were grown in a greenhouse at 28 ± 2 °C under natural light and at 25 ± 2 °C at night. Watering was performed using γ-PGA (10 kDa, 50 mg/L) solution. After growth for 30 days, the soils tightly bound to the roots (served as rhizosphere soils) were collected and analyzed for the composition of microbial community. This experiment was performed in triplicate. Amplification and high-throughput sequencing of 16 s rDNA of soil bacteria in the maize rhizosphere were performed as described by Wang et al. [64]. The primers for the V4 region of 16 s rDNA of the bacteria were 338F (5′-ACTCCTACGGGAGGCAGCA-3′) and 806R (5′-GGACTACHVGGGTWTCTAAT-3′). High-throughput sequencing was performed using the Illumina Hiseq 2000 (Illumina Inc., San Diego, USA) platform. The differences in abundance at different taxa, including the phylum, class, order, family, and genus, between groups were analyzed using Metastats. Nonmetric multidimensional scaling (NMDS) was performed on distance matrices. The 2D graphical outputs were then drawn using the coordinates. Significance difference in the relative abundance of bacteria at specific taxa was evaluated using the LEfSe analysis.

### Statistical analysis

All experiments were performed in triplicates. Continuous normally distributed data were expressed as mean ± standard deviation (SD). The difference between groups was analyzed using T-test and Duncan’s tests of one-way ANOVAs. The data were analyzed using SPSS (version 22.0.0.0). Statistical significance was set at *p < 0.05 or **p < 0.01.

## Supplementary Information

**Additional file 1: Fig. S1.** The effect of γ-PGA of different molecular weight on the maize growth and drought resistance.

**Additional file 2: Fig. S2.** The DEGs involved in auxin synthesis pathway. Roots from maize treated with γ-PGA was collected for RNA sequencing. The absolute values of log2 (CK+ γ-PGA/CK) ≥ 1, and FDR < 0.001 were used as the criteria for DEGs. The color of the box represented up (red) and down (green)-regulated genes.

**Additional file 3: Fig. S3.** The NMDS and LEfSe analysis. A, Non-metric multidimensional scaling (NMDS) for the grouping patterns of microbial communities based on the bray-curtis distance. Each colored dot represented a sample. B, LEfSe analysis (LDA ≥ 3.35) for the species in the rhizosphere soil.

**Additional file 4: Fig. S4.** The NMDS and LEfSe analysis for the species in the rhizosphere soil of the different treatment. A, Non-metric multidimensional scaling (NMDS) for the grouping patterns of microbial communities based on the bray-curtis distance. Each colored dot represented a sample. B, LEfSe analysis (LDA ≥ 3.73) for the species in the rhizosphere soil of the control maize (CK) and the maize treated with γ-PGA (CK-γ-PGA) on the normal growth condition, and the control maize (CK-D) and the maize treated with γ-PGA (CK-γ-PGA-D) after drought treatment.

**Additional file 5: Table S1.** The sequence of primers used in this study.

## Data Availability

All datasets generated for this study are included in the article/ Supplementary Materials. The data of 16 s rDNA from maize rhizosphere soil bacterial were deposited in the figshare database: https://figshare.com/articles/dataset/16s_rRNA_from_maize_rhizosphere_soil_bacterial/19127324 The data of RNAseq of the maize roots deposited in the figshare database: https://figshare.com/articles/dataset/RNA-Seq_raw_data/20000699
