# Supplementary material for: Poly-γ-glutamic acid promoted maize root development by affecting auxin signaling pathway and the abundance and diversity of rhizosphere microbial community
Source: BMC Plant Biol. 2022 Nov 10;22:521. doi: 10.1186/s12870-022-03908-y (PMC9647955; doi:10.1186/s12870-022-03908-y)
Supplement: Supplementary file 1 — Additional file 1: Fig. S1. The effect of γ-PGA of different molecular weight on the maize growth and drought resistance. [file 12870_2022_3908_MOESM1_ESM.docx]

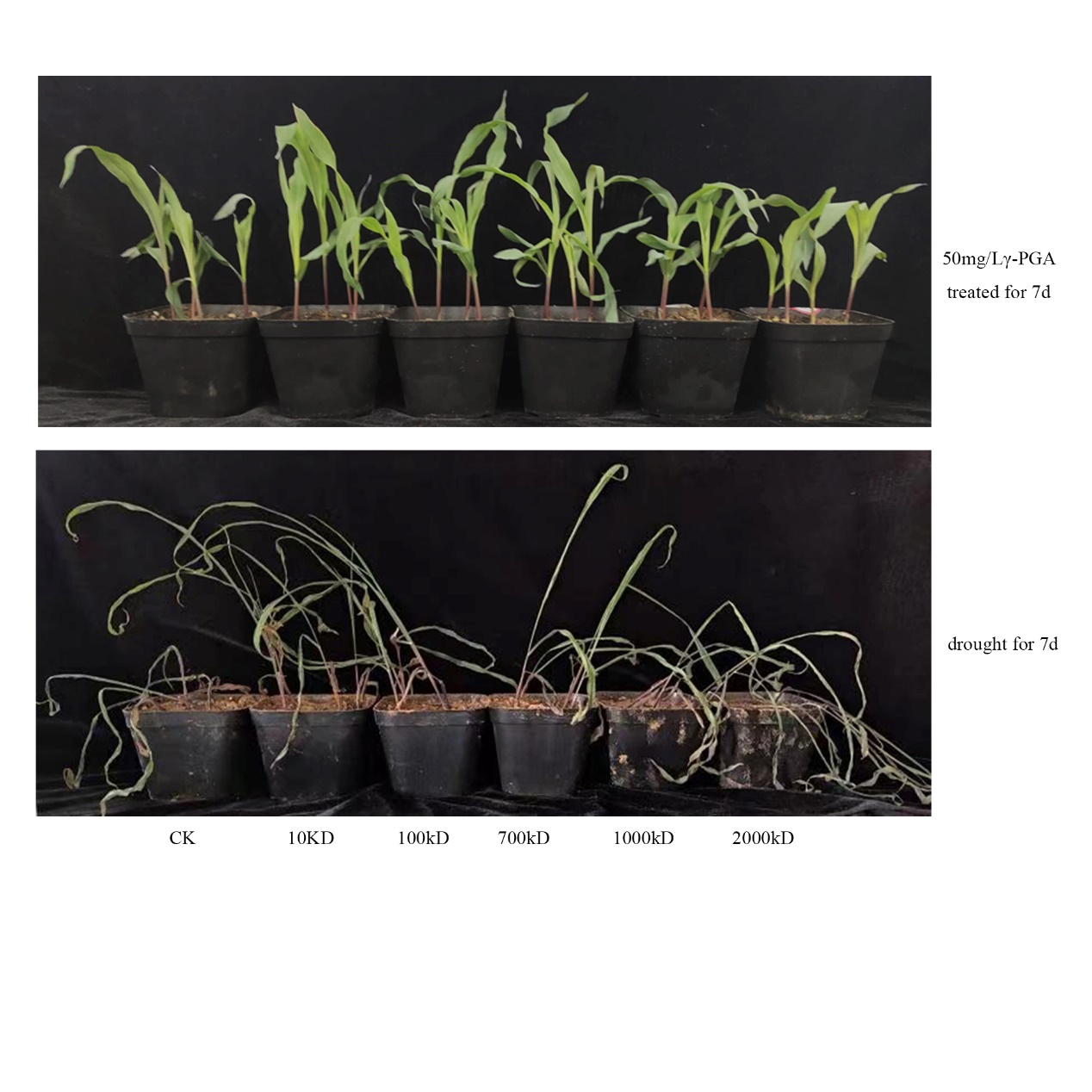


**Fig.S1 The effect of γ-PGA of** **different molecular weight on the maize growth and drought resistance**
