# Supplementary material for: Poly-γ-glutamic acid promoted maize root development by affecting auxin signaling pathway and the abundance and diversity of rhizosphere microbial community
Source: BMC Plant Biol. 2022 Nov 10;22:521. doi: 10.1186/s12870-022-03908-y (PMC9647955; doi:10.1186/s12870-022-03908-y)
Supplement: Supplementary file 2 — Additional file 2: Fig. S2. The DEGs involved in auxin synthesis pathway. Roots from maize treated with γ-PGA was collected for RNA sequencing. The absolute values of log2 (CK+ γ-PGA/CK) ≥ 1, and FDR < 0.001 were used as the criteria for DEGs. The color of the box represented up (red) and down (green)-regulated genes. [file 12870_2022_3908_MOESM2_ESM.docx]

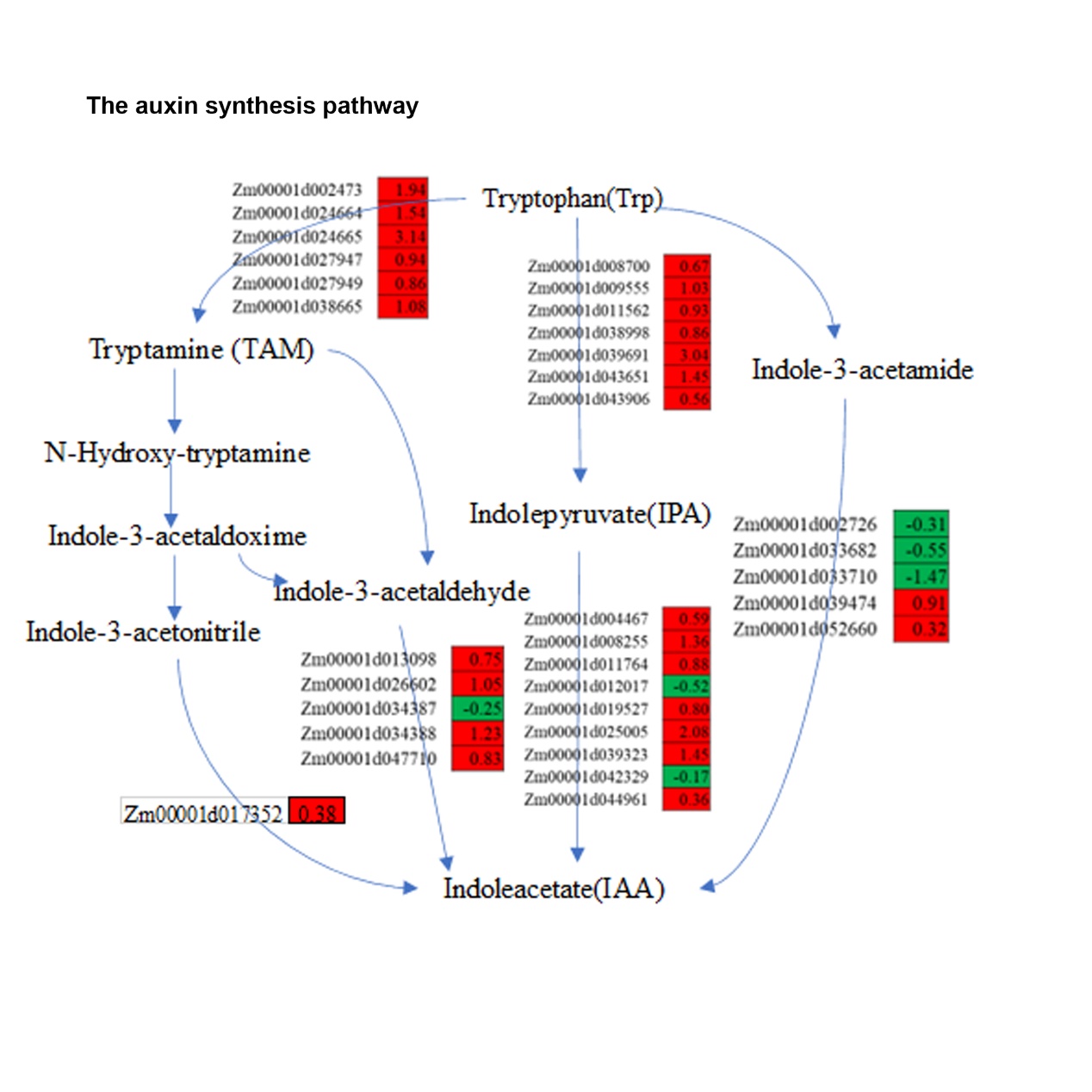


**Fig. S2. The DEGs involved in auxin synthesis pathway.**

Roots from maize treated with γ-PGA was collected for RNA sequencing. The absolute values of log2 (CK+ γ-PGA/CK)≥1, and FDR < 0.001 were used as the criteria for DEGs. The color of the box represented up (red) and down (green)-regulated genes.
