# Supplementary material for: Poly-γ-glutamic acid promoted maize root development by affecting auxin signaling pathway and the abundance and diversity of rhizosphere microbial community
Source: BMC Plant Biol. 2022 Nov 10;22:521. doi: 10.1186/s12870-022-03908-y (PMC9647955; doi:10.1186/s12870-022-03908-y)
Supplement: Supplementary file 3 — Additional file 3: Fig. S3. The NMDS and LEfSe analysis. A, Non-metric multidimensional scaling (NMDS) for the grouping patterns of microbial communities based on the bray-curtis distance. Each colored dot represented a sample. B, LEfSe analysis (LDA ≥ 3.35) for the species in the rhizosphere soil. [file 12870_2022_3908_MOESM3_ESM.docx]

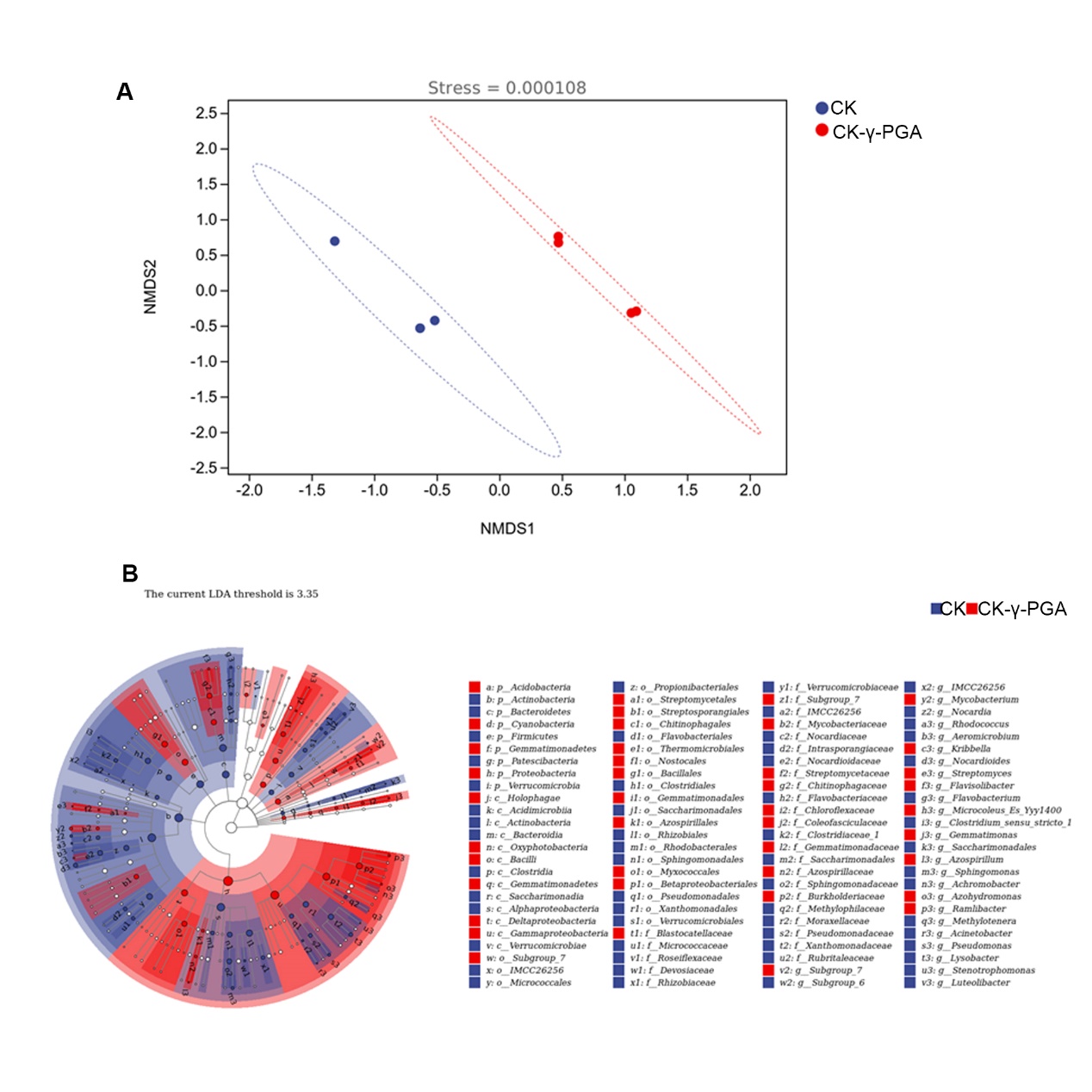


**Fig.S3. The NMDS and LEfSe analysis.**

A, Non-metric multidimensional scaling (NMDS) for the grouping patterns of microbial communities based on the bray-curtis distance. Each colored dot represented a sample. B, LEfSe analysis (LDA≥3.35) for the species in the rhizosphere soil.
