# Supplementary material for: Poly-γ-glutamic acid promoted maize root development by affecting auxin signaling pathway and the abundance and diversity of rhizosphere microbial community
Source: BMC Plant Biol. 2022 Nov 10;22:521. doi: 10.1186/s12870-022-03908-y (PMC9647955; doi:10.1186/s12870-022-03908-y)
Supplement: Supplementary file 4 — Additional file 4: Fig. S4. The NMDS and LEfSe analysis for the species in the rhizosphere soil of the different treatment. A, Non-metric multidimensional scaling (NMDS) for the grouping patterns of microbial communities based on the bray-curtis distance. Each colored dot represented a sample. B, LEfSe analysis (LDA ≥ 3.73) for the species in the rhizosphere soil of the control maize (CK) and the maize treated with γ-PGA (CK-γ-PGA) on the normal growth condition, and the control maize (CK-D) and the maize treated with γ-PGA (CK-γ-PGA-D) after drought treatment. [file 12870_2022_3908_MOESM4_ESM.docx]

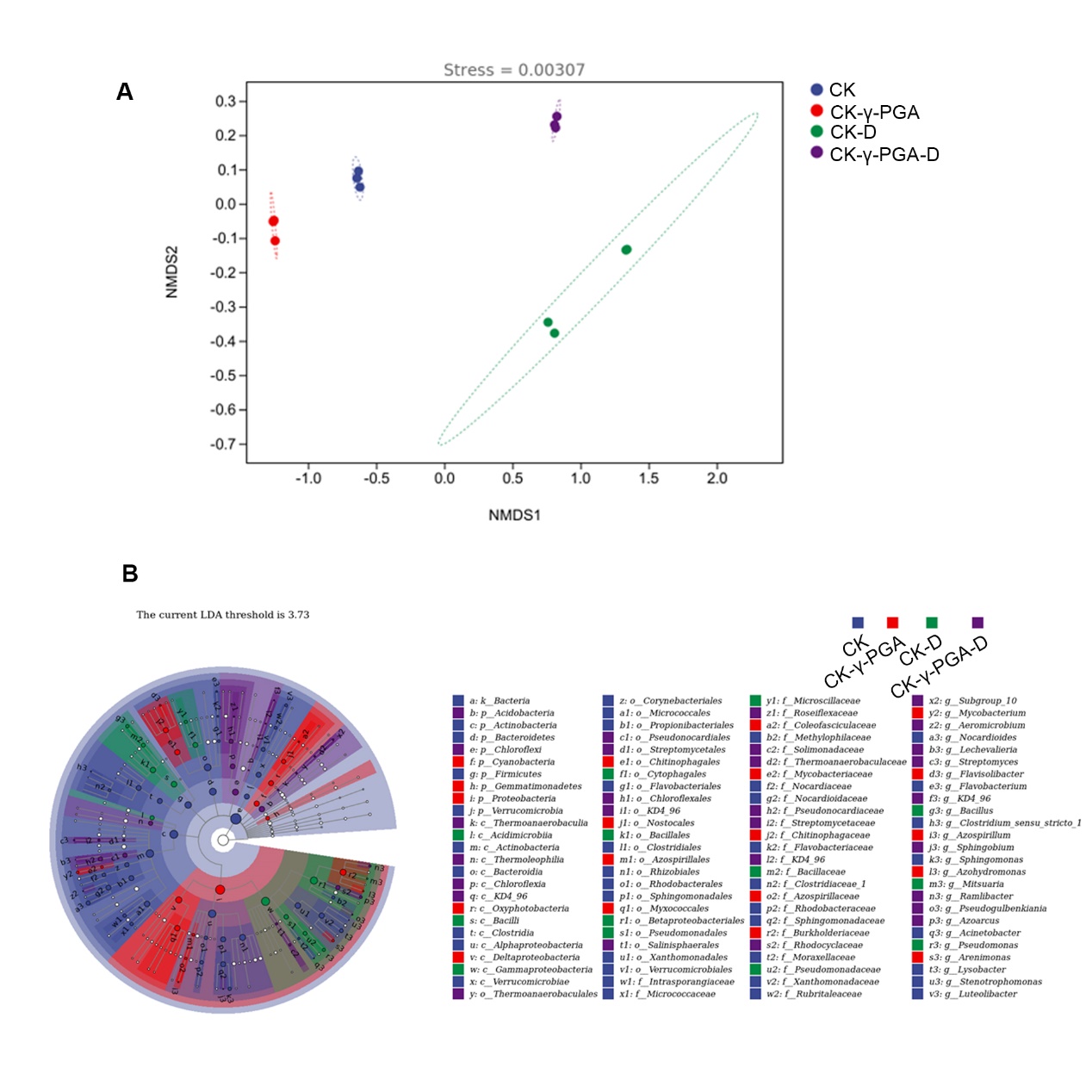


**Fig.S4. The NMDS and LEfSe analysis for the species in the rhizosphere soil of the different treatment.**

A, Non-metric multidimensional scaling (NMDS) for the grouping patterns of microbial communities based on the bray-curtis distance. Each colored dot represented a sample. B, LEfSe analysis (LDA≥3.73) for the species in the rhizosphere soil of the control maize (CK) and the maize treated with γ-PGA (CK-γ-PGA) on the normal growth condition, and the control maize (CK-D) and the maize treated with γ-PGA (CK-γ-PGA-D) after drought treatment.
